# Metabonomic Insights into the Sperm Activation Mechanisms in Ricefield Eel (*Monopterus albus*)

**DOI:** 10.3390/genes11111259

**Published:** 2020-10-26

**Authors:** Huiying Zhang, Yang Liu, Lingling Zhou, Shaohua Xu, Cheng Ye, Haifeng Tian, Zhong Li, Guangfu Hu

**Affiliations:** 1 College of Fisheries, Huazhong Agricultural University, Wuhan 430070, China; zhy_meme@163.com (H.Z.); liuyuang198310@126.com (Y.L.); llz9872@163.com (L.Z.); xsh2018308@163.com (S.X.); yechenging@163.com (C.Y.); 2 Key Lab of Freshwater Biodiversity Conservation Ministry of Agriculture, Yangtze River Fisheries Research Institute, The Chinese Academy of Fisheries Sciences, Wuhan 430223, China; tianhf@yfi.ac.cn

**Keywords:** sperm activation, metabonomics, energy metabolism, anti-oxidant stress, ricefield eel

## Abstract

In fish, sperm motility activation is one of the most essential procedures for fertilization. Previous studies have mainly focused on the external environmental effects and intracellular signals in sperm activation; however, little is known about the metabolic process of sperm motility activation in fish. In the present study, using ricefield eel (*Monopterus albus*) sperm as a model, metabonomics was used to analyze the metabolic mechanism of the sperm motility activation in fish. Firstly, 529 metabolites were identified in the sperm of ricefield eel, which were clustered into the organic acids, amino acids, nucleotides, benzene, and carbohydrates, respectively. Among them, the most abundant metabolites in sperm were L-phenylalanine, DL-leucine, L-leucine, lysolecithin choline 18:0, L-tryptophan, adenine, hypoxanthine, 7-Methylguanine, shikimic acid, and L-tyrosine. Secondly, compared to pre-activated sperm, the level of S-sulfo-L-cysteine and L-asparagine were both increased in the post-activated sperm. Ninety-two metabolites were decreased in the post-activated sperm, including quinic acid, acetylsalicylic acid, 7,8-dihydro L-biopterin, citric acid, glycylphenylalanine, and dihydrotachysterol (DHT). Finally, basing on the pathway analysis, we found that the changed metabolites in sperm motility activation were mainly clustered into energy metabolism and anti-oxidative stress. Fish sperm motility activation would be accompanied by the release of a large amount of energy, which might damage the genetic material of sperm. Thus, the anti-oxidative stress function is a critical process to maintain the normal physiological function of sperm.

## 1. Introduction

In mammals, the sperm surface is covered with many motility inhibitory factors, such as protein, polyamines, and other energy-dissipating factors, so the mammalian sperm is maintained in an inactive state when it is just ejected. After combining with some factors about capacitation in the female reproductive tract, the sperm will conduct a series of biochemical reactions and physiological changes, then acquire potential for binding the ovum [1]. Once the sperm motility is activated, the beating frequency and amplitude of the flagellum are greatly increased, which helps the sperm reach the fallopian tube [2]. As a similar result in teleost fish, the female-derived fluid surrounding the eggs (i.e., the ovarian fluid, OF) also impact sperm functional traits, such as activation, velocity, viability, longevity, and swimming trajectory [3]. In addition, the sperm of Japanese eel acquires the potential for motility activation through exposure to some factors, such as potassium, bicarbonate ion concentration, and pH in the sperm duct [4,5].

Furthermore, the acrosome reaction is another important physiological process that occurs after mammalian sperm capacitation. When the sperm gets close to the ovum, various enzymes in the acrosome are released immediately to help sperm pass through the cell membrane and complete fertilization [6]. In contrast to mammals, the fish sperms lose the acrosome, so they do not undergo acrosome reactions [7]. Previous studies have mainly focused on the seminal plasma composition and sperm regulatory signals. Firstly, several studies have reported that Na^+^, K^+^, and Cl^−^ ions play the key roles in controlling sperm motility through regulating the osmotic pressure in cyprinids and salmonids [8]. Furthermore, recent studies have also suggested that multiple signaling pathways are involved in the sperm motility activation in fish, such as protein kinase C, Ca^2+^ cascades, and cAMP/protein kinase A [9]. However, little is known about the metabolic mechanism in the sperm motility activation.

Ricefield eel (*Monopterus albus*), a protogynous hermaphrodite freshwater fish [10], has become one of the most important aquatic products in China in recent years. Before spawning, the gonads of ricefield eels are initially female ovaries, and then they transform into male testes. But this transformation limits growth, and the young female has fewer oocytes, which limits production [11]. Besides, there is also lower sperm quality in this species, and so far, the studies about sperm activation available for these natural sex reversal fish are still scarce [12]. In the present study, using ricefield eel sperm as a model, the metabolism mechanism in sperm motility activation was examined by metabonomics. This study would not only enrich the information for the mechanism in the fish sperm motility activation but also help us to improve the fertilization rate in ricefield eels.

## 2. Materials and Methods

### 2.1. Ethical Statement

In the present study, the ricefield eels were used according to the protocol approved by the committee for animal use at Huazhong Agricultural University (Ethical Approval NO. HBAC20091138; Date: 15 November 2009).

### 2.2. Sample Collection

Male ricefield eels of similar size were purchased from the local fish market and kept in well-aerated 250-litter aquaria at 28 ± 2 °C for three days. The sperms were collected from 18 ricefield eels with motility rate > 75% [13] (Figure S1). Then, the mixed sperms were divided randomly into three groups, including pre-activated group (Q), activating group (Z), and post-activated group (H). Each group had 6 parallel samples. The samples were checked under the microscope, and the defined sperm motility of the activating group (Z) was significantly weakened, and the sperm of the post-activated group (H) completely lost the motility [14]. A 100 μL of isotonic solution (NaCl, 7.8 g/L; CaCl_2_, 0.21 g/L; KCl, 0.2 g/L; NaHCO_3_, 2 g/L) was added to group Q and stored in liquid nitrogen. A 100 μL of distilled water was added to group Z for 1 min and stored in liquid nitrogen. A 100 μL of distilled water was added to group H for 3 min and stored in liquid nitrogen (Figure S2).

### 2.3. Metabolite Extraction

Samples were thawed on ice and mixed for 10 s by the vortex. Then, a 50 μL sample was placed in a 1.5 mL Eppendorf tube and mixed with 150 μL pre-chilled ice methanol (containing 1 mg/mL of 2-chlorophenylalanine as internal standard). Then, the mixed samples were vortexed for 3 min and centrifuged at 14,490× *g* at 4 °C for 10 min. The centrifuged supernatant was pipetted into another new 1.5 mL Eppendorf tube and centrifuged at 14,490× *g,* 4 °C for 5 min again. Then, the supernatant was collected into a lined tube of the sample bottle for LC-MS/MS analysis.

### 2.4. Metabolite Detection

Ultra-performance liquid chromatography (UPLC) (Shim-pack UFLC SHIMADZU CBM30A, https://www.shimadzu.com/) and tandem mass spectrometry (QTRAP^®^, https://sciex.com/) were used to analyze the chromatograms and mass spectra of the sample extracts. The liquid phase conditions were listed as follows: UPLC: column, Waters ACQUITY UPLC HSS T3 C18 (1.8 µm, 2.1 mm × 100 mm); solvent system, ultrapure water (0.04% acetic acid): acetonitrile (0.04% acetic acid); gradient program, 95:5 *V/V* at 0 min, 5:95 *V/V* at 11.0 min, 5:95 *V/V* at 12.0 min, 95:5 *V/V* at 12.1 min, 95:5 *V/V* at 14.0 min; flow rate, 0.4 mL/min; column temperature, 40 °C; injection volume 2 μL [15].

Mass spectrometry operation parameters were as follows: electrospray ionization (ESI) temperature 500 °C; mass spectrometry voltage 5500 V (positive), −4500 V (negative); ion source gas I (GSI), gas II (GSII), curtain gas (CUR) were set at 55, 60, 25 psi, respectively; the collision-activated dissociation (CAD) was set to high. Each ion-pair was scanned according to the optimized declustering potential (DP) and collision energy (CE) in the triple quadrupole (Q trap) [16].

### 2.5. Data Analysis

Based on the self-built target Metware Database (MWDB) of Metware Biotechnology Co., Ltd. (Wuhan, China), qualitative analysis was performed according to the retention time (RT), ion-pair information, and secondary spectrum data. Quantitative analysis of metabolites was performed by the multiple reaction monitoring mode (MRM) of triple quadrupole mass spectrometry. Analyst 1.6.3 (AB SCIEX, Foster City, CA, USA) was used to process mass spectrometry data. R3.5.1 programming language (R Foundation for Statistical Computing, Vienna, Austria) was used for data processing and analyses by the unit-variance algorithm. Principal Components Analysis (PCA) was performed on the data of the R prcomp package, and Orthogonal signal correction and Partial Least Squares-Discriminant Analysis (OPLS-DA (Orthogonal signal correction and Partial Least Squares-Discriminant Analysis)) was executed by the R opls software package. The differentially expressed metabolites were screened out by the value of variable importance in projection (VIP) and fold change (FC) (VIP > 1, FC > 2, or FC < 0.5). Kyoto Encyclopedia of Genes and Genomes (KEGG (Kyoto Encyclopedia of Genes and Genomes)) database was used to annotate the corresponding pathways formed by the interaction of differentially expressed metabolites in the organism. 

## 3. Results

### 3.1. Composition Analysis of the Sperm in Ricefield Eels

**Figure 1:**
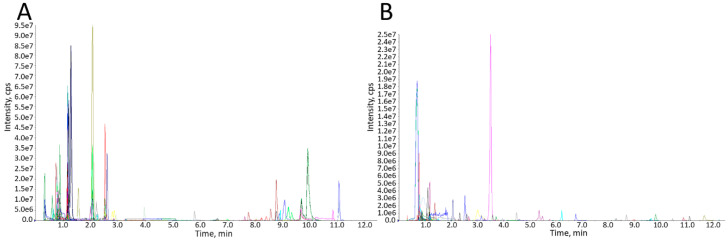
Multimodality of MRM metabolite detection. Chromatographic peaks of different colors represent different metabolites detected. The triple quadrupole was used to screen the characteristic ions of each metabolite detected, and the CPS (counts per second) of characteristic ions was obtained; then, the peak area of each chromatographic peak was corrected and integrated; thus, the size of the area represented the relative content of corresponding metabolites. (**A**) Positive ion detection, (**B**) Negative ion detection. MRM, multiple reaction monitoring mode.

**Figure 2:**
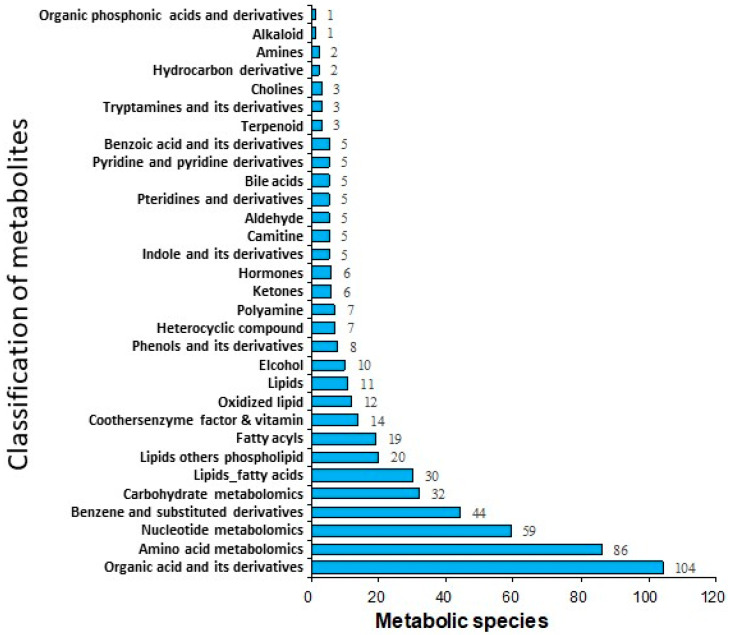
Composition of the metabolites in the pre-activated ricefield eel *(Monopterus albus)* sperm.

As shown in Figure 1, 529 metabolites were identified in the pre-activated sperm. Among them, there were 104 organic acids and their derivatives, 86 amino acids and their derivatives, 59 nucleotides and their derivatives, 44 benzene and its derivatives, 32 carbohydrates and their metabolites, and 30 free fatty acids (Figure 2). The top 19 metabolites with high levels in the pre-activated sperm are listed in Table 1, including L-phenylalanine, DL-leucine, L-leucine, lyso-phosphatidylcholine (PC), L-tryptophan, adenine, hypoxanthine, 7-methylguanine, shikimic acid, L-tyrosine, choline chloride, L-valine, 2-nonanone, cis-3-hexenylacetate, nicotinamide, methylcysteine, acetyl-L-carnitine, L-glutamic acid, and oleamide. 

### 3.2. Principal Component Analysis (PCA)

**Figure 3:**
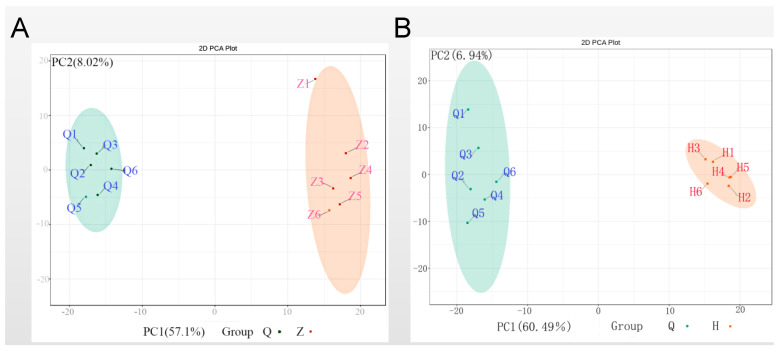
Principal component analysis comparing pre-activated sperm (Q) towards either (**A**) activating sperm (Z) or (**B**) post-activated sperm (H).

To provide insights into separations among these experimental groups based on the chromatograms and mass spectra measurements from analytical instrumentation, PCA was performed to compare the differences among the pre-activated group, activating group, and post-activated group. As shown in Figure 3, the three groups were segregated into a tight cluster in this unsupervised model. But significant differences were observed not only between the pre-activated group and the activating group but also between the pre-activated group and the post-activated group. The PC1 weights of the above two sets of results accounted for 60.49% and 57.1%, respectively. In addition, there was no significant difference between the activating group and the post-activated group.

### 3.3. Orthogonal Projections to Latent Structures-Discrimination Analysis (OPLS-DA)

**Figure 4:**
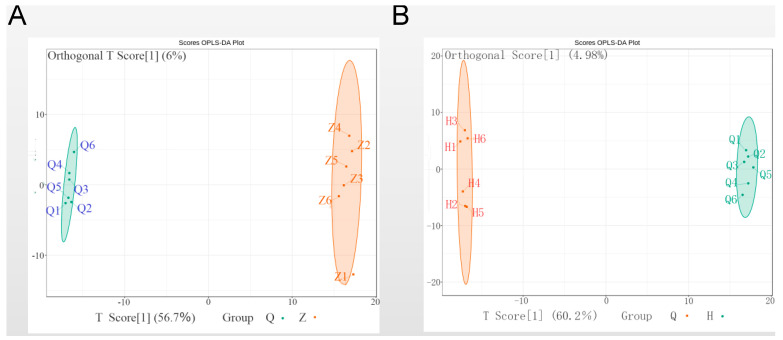
Orthogonal projections to latent structures-discrimination analysis (OPLS-DA) comparing pre-activated sperm (Q) towards either (**A**) activating sperm (Z) or (**B**) post-activated sperm (H).

Orthogonal projections to latent structures-discrimination analysis (OPLS-DA) model was established to aggressively force discrimination between experimental groups and to improve the validity and reliability of the result. In Figure 4, this supervised model gave the orthogonal T score of 56.7% and 60.2% to distinguish the samples of the pre-activated group and activating group, pre-activated group and post-activated group, respectively, which further confirmed that there were significant discriminations between these two pair of groups. 

### 3.4. Differential Metabolite Analysis in the Sperm Motility Activation

**Figure 5:**
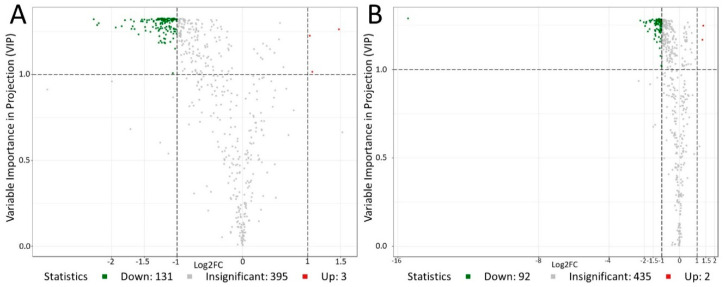
Volcano map of differential metabolites between the pre-activated sperm and activating sperm (**A**), pre-activated sperm and post-activated sperm (**B**). Each point in the figure represents a metabolite. Green dots represent down-regulated metabolites, so the number of down-regulated metabolites in A and B is 131 and 92, respectively. Red dots represent up-regulated metabolites, so the number of up-regulated metabolites in A and B is 3 and 2, respectively. Grey dots represent identified but insignificantly different metabolites.

After combining the fold change with the VIP value for screening, the differential metabolites statistics in the three stages of sperm motility activation are shown in Figure 5. Compared to the pre-activated sperm, there were 131 down-regulated metabolites and three up-regulated metabolites (glutathione reduced form, L-asparagine anhydrous, S-sulfo-L-cysteine) in activating sperm (Table 2). In addition, there were 94 differential metabolites between the pre-activated sperm and post-activated sperm, including 92 down-regulated and two up-regulated metabolites (L-asparagine anhydrous, S-sulfo-L-cysteine) in the post-activated sperm compared to the pre-activated sperm (Table 2). 

Compared with the pre-activated sperm, the up-regulated differential metabolites in the post-activated sperm included S-sulfo-L-cysteine and L-asparagine (Table 3). The top down-regulated differential metabolites were quinic acid, acetylsalicylic acid, 7,8-dihydro L-biopterin, citric acid, glycylphenylalanine, dihydrotachysterol (Table 3). These two up-regulated metabolites were both amino acids and their derivatives, and 92 down-regulated metabolites included 35 organic acids and their derivatives, 13 amino acids and their derivatives, 11 nuclear glycosides and their metabolites, and five benzene and its derivatives (Table 3).

### 3.5. KEGG Pathway Analysis for Differential Metabolites

**Figure 6:**
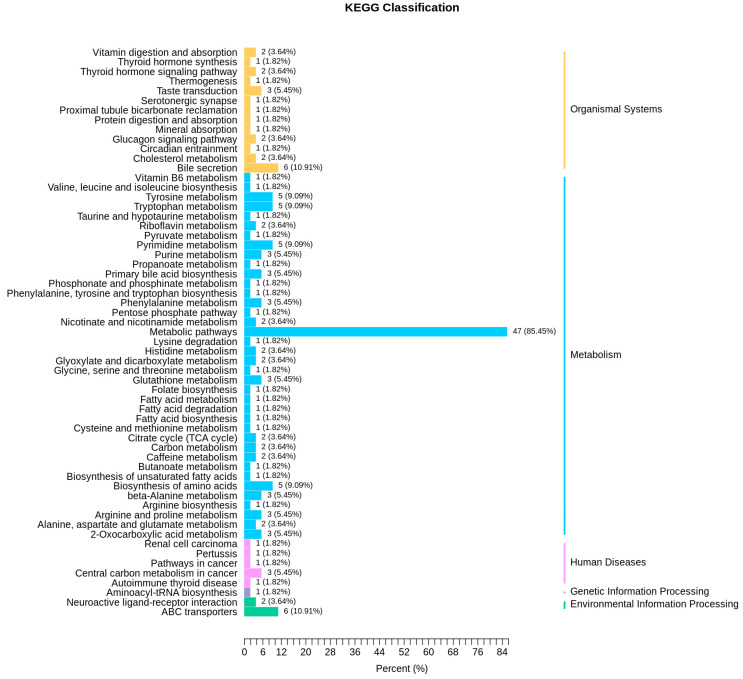
Kyoto Encyclopedia of Genes and Genomes (KEGG) classification of differential metabolites between the pre-activated and post-activated sperm. The annotated metabolic pathways are classified according to the pathway type in KEGG. The abscissa is the number of metabolites annotated to this pathway and the ratio of the number of metabolites annotated in the corresponding pathway to the total number of annotated metabolites.

**Figure 7:**
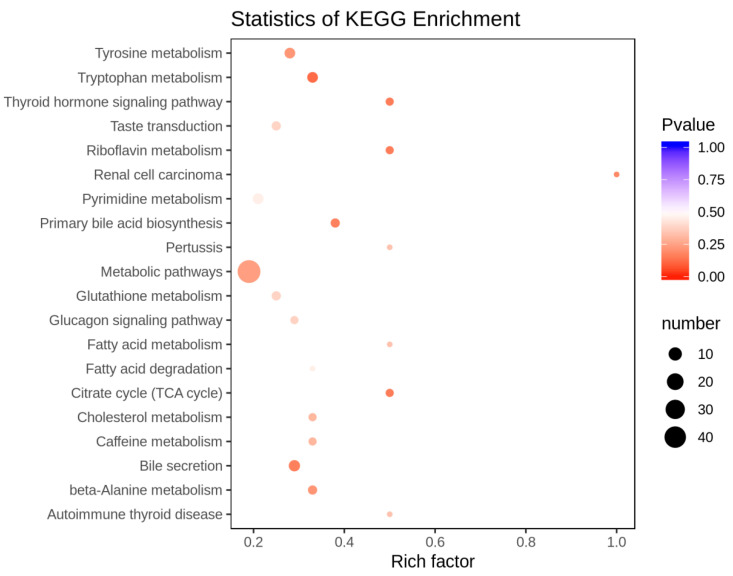
KEGG pathway enrichment analysis for differential metabolites between the pre-activated and post-activated sperm. The rich factor is the ratio of the number of differential metabolites in metabolic pathways to the total number of metabolites.

KEGG (Kyoto Encyclopedia of Genes and Genomes) database is a powerful tool for biological metabolism analysis and metabolic network research. In the present study, KEGG analysis was used to annotate differential metabolites. Compared to the pre-activated sperm, two up-regulated metabolites (L-asparagine anhydrous and S-sulfo-L-cysteine) in the post-activated sperm were annotated on the amino acid biosynthetic pathway. In addition, the down-regulated metabolites in the pre-activated sperm were mainly annotated on several metabolic pathways, including bile secretion, ABC transporter, tyrosine metabolism, tryptophan metabolism, pyrimidine metabolism, amino acid biosynthesis, and glutathione metabolism (Figure 6). Furthermore, KEGG enrichment analysis showed that these differential metabolites between the pre-activated and post-activated sperm were enriched mainly in metabolic pathways, bile secretion, tryptophan metabolism, primary bile acid biosynthesis, citrate cycle, and beta−alanine metabolism (Figure 7).

## 4. Discussion

### 4.1. Composition Analysis of Metabolites in Ricefield Eel Sperm

Metabonomics can reveal the downstream events of gene expression, so it is more closely related to actual phenotypes than transcriptomics. The main components of metabolites that are identified in ricefield eel sperm include organic acids and their derivatives, amino acids and their derivatives, nucleotides and their derivatives, benzene and their derivatives, carbohydrates and their metabolites, and lipids. This finding is consistent with the result in the human sperm metabolome [17]. However, the metabolites types in ricefield eel sperm are more abundant than that in human sperm, and there are also differences in the content of these metabolites. Amino acids with their derivatives and carnitine metabolites account for the main components in human sperm [18]; in contrast, organic acids with their derivatives and amino acids with their derivatives account for the major components in ricefield eel sperm. Similarly, certain amino acids regulate milt biochemistry and male ejaculate traits in European eel, *Anguilla anguilla* [19]. Thus, we found that the carnitine content in ricefield eel sperm was lower than that in the human sperm.

It has been reported that free amino acids could be used as the chelating agents (especially for toxic metals) or the oxidizable substrate for sperm [20]. It can also be catabolized by transamination, decarboxylation, and oxidative deamination. The amino acid fragments are theoretically used as fuel, mainly to provide energy through the TCA cycle, or as the basis for various biosynthetic processes. The important raw materials for ATP synthesis are nucleotides and their derivatives, and the expression level of cAMP is regulated by the activation of adenosine receptors on the sperm surface. They are both critical for sperm capacitation [21]. The energy derived from carbohydrate metabolism in mitochondria is a vital source of mammalian sperm motility, and some evidences have demonstrated that glycolysis could compensate for the lack of oxidative phosphorylation and restore most functions of sperm [22]. Lipid is a crucial component of the biomembrane system; thus, lipid raft movement is one of the critical steps to complete the mice sperm maturation [23]. Previous studies have indicated that reducing lipid metabolism will significantly inhibit sperm motility [24]. Carnitine is usually a product of fatty acid metabolism, and it not only plays a role in sperm maturity but also affects the energy metabolism of sperm [25]. In addition, it may also be used as an anti-apoptotic and antioxidant factor [26]. 

### 4.2. Analysis of Up-Regulated Metabolites in the Post-Activated Sperm 

Reactive oxygen species (ROS) is inevitable during energy metabolism in sperm. It has been found that superoxide is able to promote human sperm capacitation [27]; however, excessively high concentrations of ROS can reduce sperm fertility [28]. Previous studies have suggested that elevated ROS could induce the formation of lipids in neurons that are transferred to glia where they form lipid droplets (LD), and furthermore, ROS and LD accumulation are the primary contributors to cell death [29]. Fortunately, cysteine can reduce the damage of excessive ROS to sperm plasma membrane, DNA, and mitochondria [30], and methionine can increase the antioxidant capacity of sperm [31]. S-sulfo-L-cysteine is derived from the metabolism of cysteine and methionine. Thus, in the present study, the increase in S-sulfo-L-cysteine content in the post-activated sperm might be the result of sperm self-protection, which is to reduce the damage caused by the large amounts of ROS produced during activation.

### 4.3. Analysis of Down-Regulated Metabolites in the Post-Activated Sperm

Quinic acid is a cyclohexane formic acid that is widely distributed in plants. Previous studies have suggested that quinic acid plays an important role in anti-diabetic [32] and anti-neuritis [33]. Further studies have found that it may also be used as a powerful antioxidant, which can be caused by its conversion into the compound that can mediate antioxidant effects [34]. Excessive oxidation is an unavoidable threat in sperm activation. In the present study, the content of quinic acid was greatly reduced in the post-activated sperm and was even almost completely consumed. This phenomenon might be related to the effects of quinic acid on anti-oxidative stress.

As we know, glycolysis is the most common oxidative energy supply in the body, and it is no exception during sperm activation. A previous study has reported that the metabolic inhibitors, substrates, coenzymes, and oxygen concentrations could influence the sperm motility in teleost [35]. Similar to our study, these results have shown that the metabolites related to oxidative phosphorylation, tricarboxylic acid cycle, and aerobic glycolysis are central energy-supplying pathways for the sperm of fish. Furthermore, the most crucial intermediate product of the TCA cycle is citric acid, whose decrease in the post-activated sperm might be caused by the increase in the TCA cycle, which results from the consumption of a large amount of mitochondrial ATP during sperm activation.

Previous studies have shown that spermidine may restrain sperm capacitation by inhibiting the lateral diffusion of proteins in the plasma membrane of mammalian sperm, and heparin in the reproductive tract of female animals can relieve its inhibitory effect on sperm by removing spermidine in the seminal plasma [36]. Recently, a study has indicated that spermidine can prevent heart injury in rats exposed to hypoxia by inhibiting oxidative stress [37]. So, we assumed that in the post-activated ricefield eel sperm, the spermidine content of sperm dropped rapidly, which might be due to its redox reaction with certain substances in water and being consumed.

### 4.4. KEGG Pathway Analysis

ATP-binding cassette (ABC) transporter is an ATP-dependent transmembrane transporter, which is related to the transmembrane transport of multiple compounds or ions. As we know, one of the features of the initial stage in sperm capacitation is the release of large amounts of cholesterol from the plasma membrane. Previous studies have shown that ABC transporter is involved in the transport of endogenous lipids and plays an important role in sterol efflux [38]. The reason why the capacitation reaction can proceed smoothly may be that the sterol efflux makes the decapacitation factor detach from the surface [39], so this assumption is consistent with our results in KEGG classification. 

Glutathione metabolism is one of the major metabolic pathways in our result of KEGG classification. Owing to the presence of a large amount of unsaturated fatty acids, the sperm plasma membrane is easily destroyed by ROS. Luckily, there is an important antioxidant or called free radical scavenger named glutathione in the body. Recent studies have revealed that it is effective to significantly improve the fertilization ability of bovine sperm by adding glutathione to seminal plasma [40]; this result also improves the reliability of our study.

## 5. Conclusions

In the present study, by using metabonomic technology, we examined the metabolic mechanism of sperm motility activation in the ricefield eel. Firstly, the compositional differences between the sperm metabolites of ricefield eel and human sperm were analyzed, and the results indicated that the metabolite types in the ricefield eel sperm were more abundant than that in humans. Secondly, compared to the pre-activated sperm, the levels of S-sulfo-L-cysteine and L-asparagine were both increased in the post-activated sperm. However, the content of quinic acid and spermidine was greatly reduced in the post-activated sperm compared to the pre-activated sperm. Finally, basing on the pathway analysis, we found that the changed metabolites in sperm motility activation were also mainly clustered into energy metabolism and anti-oxidative stress. These results suggested that sperm activation in teleost not only involved the mitochondrial energy metabolism but also the anti-oxidative stress system to prevent sperm from being damaged.

## Figures and Tables

**Table 1:** The highest content of metabolites in the pre-activated sperm (TOP19).

Compounds	Class	Average Content (CPS)
L-phenylalanine	Amino acid metabolomics	374,765,000.00
DL-leucine	Amino acid metabolomics	316,850,000.00
L-leucine	Amino acid metabolomics	312,960,000.00
LysoPC	Lipids others phospholipid	305,792,833.33
L-tryptophan	Amino acid metabolomics	165,811,666.67
Adenine	Nucleotide metabolomics	161,918,333.33
Hypoxanthine	Nucleotide metabolomics	144,711,666.67
7-methylguanine	Nucleotide metabolomics	132,558,333.33
Shikimic acid	Organic acid and its derivatives	130,220,000.00
L-tyrosine	Amino acid metabolomics	122,846,666.67
Choline chloride	Others	116,896,666.67
L-valine	Amino acid metabolomics	111,735,000.00
2-nonanone	Ketones	101,428,333.33
Cis-3-hexenylacetate	Fatty acyls	97,867,000.00
Nicotinamide	Co others enzyme factor and vitamin	72,400,500.00
Methylcysteine	Amino acid metabolomics	70,355,333.33
Acetyl-L-carnitine	Camitine	70,314,000.00
L-glutamic acid	Amino acid metabolomics	69,354,000.00
Oleamide	Lipids fatty acids	68,706,833.33

**Table 2:** Statistics of the number of differential metabolites.

Group Name	Total Sig Metabolites	Down-Regulated	Up-Regulated
Q vs. Z	134	131	3
Z vs. H	1	1	0
Q vs. H	94	92	2

**Table 3:** Different metabolites between the pre-activated sperm (Q) and post-activated sperm (H).

Metabolites	Class	VIP Value	FC (H/Q)	Type
S-sulfo-L-cysteine	Amino acids	1.25	2.56	up
L-asparagine anhydrous	Amino acids	1.17	2.47	up
Qinic acid	Organic acid	1.29	2.29 × 10^5^	down
Aspirin	Organic acid	1.28	0.21	down
7,8-dihydro-L-biopterin	Pteridines	1.27	0.25	down
Citric acid	Amino acid metabolomics	1.25	0.26	down
Glycylphenylalanine	Amino acid metabolomics	1.26	0.27	down
Dihydrotachysterol	Hormones	1.27	0.27	down
Taurochenodesoxycholic acid	Bile acids	1.22	0.29	down
1-methyluric acid	Organic acid	1.28	0.31	down
Dl-glyceraldehyde-3-phosphate	Organic acid	1.23	0.31	down
N-acetyl-L-glutamic acid	Amino acid metabolomics	1.27	0.32	down
Isoquinoline	Benzene and substituted derivatives	1.28	0.33	down
Indoxyl sulfuric acid	Organic acid	1.28	0.35	down
Flavin adenine dinucleotide	Nucleotide metabolomics	1.17	0.37	down
Furfural	Organic acid	1.28	0.37	down
Spermidine	Polyamine	1.26	0.38	down
3,3′,5-triiodo-L-thyronine	Hormones	1.22	0.38	down
Nicotinic acid	Co others enzyme Factor	1.20	0.38	down
Inosine diphosphate (IDP)	Nucleotide metabolomics	1.22	0.39	down
Palmitoylcarnitine	Camitine	1.25	0.39	down
Hydroquinone	Phenols	1.19	0.39	down

Noted: VIP, variable importance in projection; FC, fold change.

## Supplementary Materials

The following are available online at https://www.mdpi.com/2073-4425/11/11/1259/s1, Figure S1: Semen collection from the ricefield eels (*Monopterus albus*), Figure S2: The experimental design model.
