# Supplementary material for: Metabonomic Insights into the Sperm Activation Mechanisms in Ricefield Eel (Monopterus albus)
Source: Genes (Basel). 2020 Oct 26;11(11):1259. doi: 10.3390/genes11111259 (PMC7692440; doi:10.3390/genes11111259)
Supplement: Supplementary file 1 [file genes-11-01259-s001.zip › genes-978839-supplementary.pptx]

## Slide 1
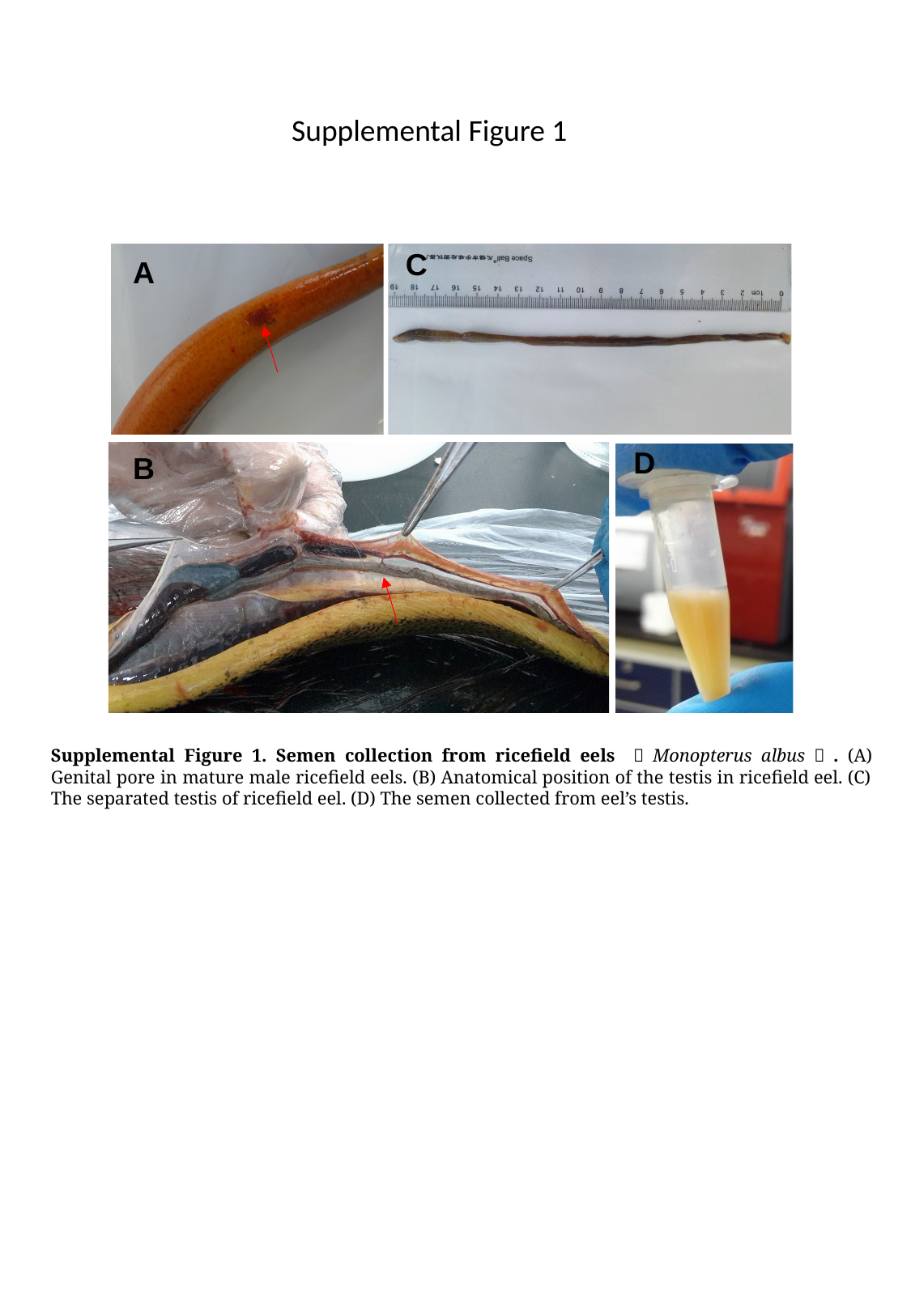

Supplemental Figure 1
C
A
D
B
Supplemental Figure 1. Semen collection from ricefield eels （Monopterus albus）. (A) Genital pore in mature male ricefield eels. (B) Anatomical position of the testis in ricefield eel. (C) The separated testis of ricefield eel. (D) The semen collected from eel’s testis.

## Slide 2
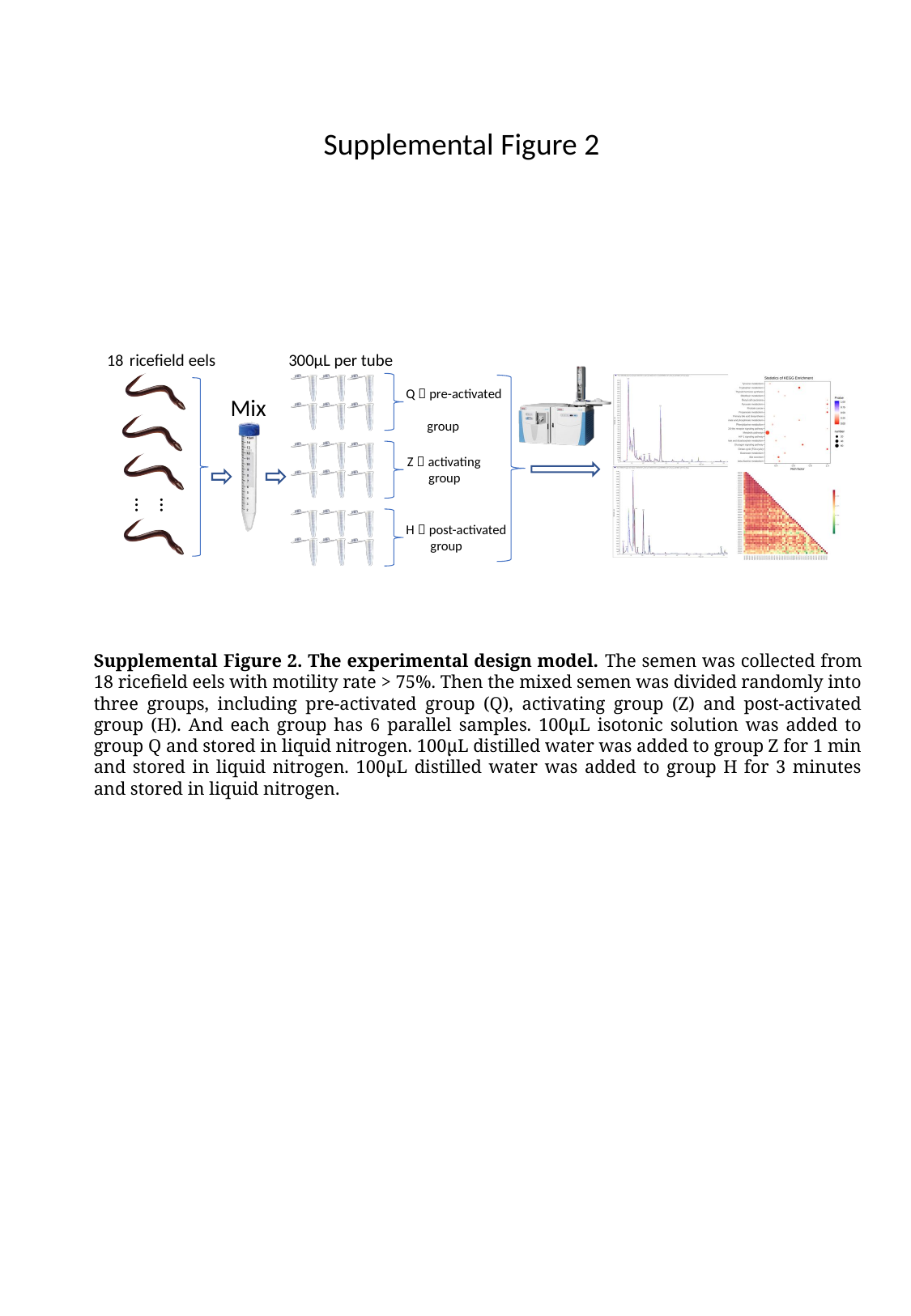

Supplemental Figure 2
ricefield eels
300μL per tube
18
Q：pre-activated
 group
Mix
Z：activating
 group
……
H：post-activated
 group
Supplemental Figure 2. The experimental design model. The semen was collected from 18 ricefield eels with motility rate > 75%. Then the mixed semen was divided randomly into three groups, including pre-activated group (Q), activating group (Z) and post-activated group (H). And each group has 6 parallel samples. 100μL isotonic solution was added to group Q and stored in liquid nitrogen. 100μL distilled water was added to group Z for 1 min and stored in liquid nitrogen. 100μL distilled water was added to group H for 3 minutes and stored in liquid nitrogen.
